# Phenobarbital reduces EEG amplitude and propagation of neonatal seizures but does not alter performance of automated seizure detection

**DOI:** 10.1016/j.clinph.2016.07.007

**Published:** 2016-10

**Authors:** Sean R. Mathieson, Vicki Livingstone, Evonne Low, Ronit Pressler, Janet M. Rennie, Geraldine B. Boylan

**Affiliations:** a Academic Research Department of Neonatology, Institute for Women’s Health, University College London, London, United Kingdom; b Neonatal Brain Research Group, Irish Centre for Fetal and Neonatal Translational Research and Department of Paediatrics and Child Health, University College Cork, Ireland; c Department of Clinical Neurophysiology, Great Ormond Street Hospital, London, United Kingdom

**Keywords:** Electroclinical uncoupling, Neonatal seizures, Automated seizure detection

## Abstract

**Objective:**

Phenobarbital increases electroclinical uncoupling and our preliminary observations suggest it may also affect electrographic seizure morphology. This may alter the performance of a novel seizure detection algorithm (SDA) developed by our group.

The objectives of this study were to compare the morphology of seizures before and after phenobarbital administration in neonates and to determine the effect of any changes on automated seizure detection rates.

**Methods:**

The EEGs of 18 term neonates with seizures both pre- and post-phenobarbital (524 seizures) administration were studied. Ten features of seizures were manually quantified and summary measures for each neonate were statistically compared between pre- and post-phenobarbital seizures. SDA seizure detection rates were also compared.

**Results:**

Post-phenobarbital seizures showed significantly lower amplitude (*p* < 0.001) and involved fewer EEG channels at the peak of seizure (*p* < 0.05). No other features or SDA detection rates showed a statistical difference.

**Conclusion:**

These findings show that phenobarbital reduces both the amplitude and propagation of seizures which may help to explain electroclinical uncoupling of seizures. The seizure detection rate of the algorithm was unaffected by these changes.

**Significance:**

The results suggest that users should not need to adjust the SDA sensitivity threshold after phenobarbital administration.

## Highlights

• Phenobarbital reduces both amplitude and propagation of neonatal seizures.
• These changes may help to explain electroclinical uncoupling.
• The performance of our seizure detection algorithm was unaffected.

## 1 Introduction

Seizures are common in the neonatal period but clinical diagnosis of seizures is imprecise (Murray et al., 2008). EEG remains the only reliable method for detecting all neonatal seizures. It is common practice to monitor neonates with amplitude integrated EEG (aEEG) for long periods, particularly those undergoing therapeutic hypothermia, whereas continuous EEG (cEEG) is available in only very few neonatal units. There is often a lack of trained neurophysiology experts available to review the EEG and identify seizures. To meet this need, a novel automated seizure detection algorithm, the **A**lgorithm for **N**eonatal **S**eizure **R**ecognition (ANSeR), has been developed by our group (Temko et al., 2011, Mathieson et al., 2016b).

Phenobarbital remains the primary first-line treatment for neonatal seizures and exerts its primary inhibitory effect by prolonging and potentiating the action of GABA on the GABA_A_ receptor, hyperpolarising neurons, although there is evidence that the AMPA/kainate subtype of glutamate receptor may also be blocked by phenobarbital (Nardou et al., 2011, Loscher and Rogawski, 2012). Studies have shown that its effectiveness is limited and seizures are abolished in only 30–50% of cases (Painter et al., 1999, Boylan et al., 2002, Boylan et al., 2004). In neonates with severe encephalopathy, seizures can often be intractable to phenobarbital, characterized by significantly abnormal background EEG patterns and high seizure burden. ‘Electroclinical dissociation’ is a term that has been variously used to describe clinical seizures without an EEG correlate (Weiner et al., 1991) or conversely any electrical seizures without a clinical correlate (Boylan et al., 2002). The term ‘electroclinical uncoupling’ has been used to describe the loss of the clinical component of an electroclinical seizure after anticonvulsant administration, resulting in ongoing electrographic seizures (Scher et al., 2003, Glykys et al., 2009), and is the term preferred here to describe changes in seizures after phenobarbital. Phenobarbital and other anticonvulsants are known to cause electroclinical uncoupling. In many neonates, electroclinical seizures become purely electrographic after treatment with phenobarbital or phenytoin. In one study (Scher et al., 2003) it was found that 58% of neonates who had electroclinical seizures before anticonvulsant administration went on to have “electrographic only” seizures after treatment. In another study incorporating data on seizure burden (Boylan et al., 2002), ten neonates showed a significant decrease in clinical seizures after medication but showed persisting electrographic seizures with an increasing seizure burden in some neonates. Quite often seizures appear to reduce in amplitude (personal observations) after phenobarbital which has not been reported in the scientific literature and may be a contributing factor to the electroclinical uncoupling of seizures in neonates.

In assessing the performance of seizure detection algorithms, (e.g. ANSeR), engineers use general performance metrics such as seizure detection and false detection rates or sensitivity and specificity based on epoched data. End users also wish to know specifically what type of seizures the algorithm detects best and in a previous study (Mathieson et al., 2016a), a novel in-depth methodology, using 10 criteria to quantify seizures, was developed to determine the ‘features’ of detected and non-detected seizures. False detections were also categorized. The results facilitated targeted improvements to the algorithm (Temko et al., 2013). End-users also wish to know how a seizure detection algorithm will perform under specific circumstances such as after administration of anticonvulsants, as any drop in performance may delay further seizure identification and affect appropriate titration of medication. This issue has not been discussed in previous performance analysis of other seizure detection algorithms (Gotman et al., 1997, Altenburg et al., 2003, Aarabi et al., 2006, Navakatikyan et al., 2006, Deburchgraeve et al., 2008, Mitra et al., 2009).

The aims of this study were to examine specifically how phenobarbital affects the morphology of neonatal seizures using our ‘in-depth’ analysis methodology, and whether the performance of our own algorithm, ANSeR, is altered. Changes in seizure detection rates due to anticonvulsants may affect how users set the variable sensitivity thresholds available to the ANSeR end-user.

## 2 Methods

### 2.1 Patients and EEG recording

The EEGs of 18 term neonates who underwent continuous EEG monitoring at the neonatal units at University College Hospital, London and University College Hospital, Cork between November 2009 and February 2012 were extracted from our database of recordings. Only neonates with EEG seizures both pre- and post-phenobarbital administration were included. As the number of post-phenobarbital seizures generally greatly exceeded the number of pre-phenobarbital seizures, for statistical purposes, pre- and post-phenobarbital seizure numbers were matched by taking the smaller number of seizures pre- or post-phenobarbital administration. For example, if a neonate had only 5 seizures before the first dose of phenobarbital and 20 after, then only the first 5 seizures pre-phenobarbital and the first 5 seizures post-phenobarbital administration were analysed.

Neonates underwent video-EEG monitoring using the NicoletOne ICU monitor (Carefusion, Wisconsin, USA) for as long as clinically required. A sampling frequency of 250 Hz or 256 Hz was used with a filter bandwidth of 0.5–70 Hz and a 50 Hz notch filter. Recording electrodes were applied using the 10:20 measuring system adapted for neonates and included F4, F3, T4, C4, CZ, C3, T3, O2 and O1 which were displayed in a bipolar montage. ECG from shoulder electrodes and respiration from a motion sensor on the abdomen were also recorded in the trace. This reduced montage is our standard clinical setup and is used to balance the requirement to detect the majority of seizures against the need to minimise handling in sick neonates who are often unstable. A study by Tekgul (Tekgul, 2005) demonstrated a sensitivity of 96.8% for a 9 electrode montage, similar to ours, to detect seizures compared to the full 10/20 montage. While this montage may not have detected the full field of seizures in this study, we did not anticipate that this should affect the result unduly, as the same montage was used to record both pre- and post-phenobarbital seizures.

All babies had an MRI performed to clarify the underlying brain pathology except in the occasional severe case where the patient unfortunately died very early in the period of intensive care.

### 2.2 Ethics and consent

This study was approved by the East London and the City Research Ethics Committee 09/H0703/97 and by the Clinical Research Ethics Committees of the Cork Teaching Hospitals. Written informed consent was obtained from one parent of each neonate who participated in the study.

### 2.3 Seizure detection algorithm

A detailed description of ANSeR is described by Temko (Temko et al., 2011). To summarize, in a preprocessing step, the original EEG undergoes artefact removal (simple high frequency artefacts are removed by applying a threshold to the signal energy), then is downsampled to 32 Hz with an anti-aliasing filter at 12.8 Hz and then segmented into 8 s epochs with a 50% overlap. Fifty five ‘features’ of the EEG are then extracted for each channel and from each epoch which can be grouped into time domain characteristics (eg. root mean squared amplitude, autoregressive error modelling, variance), frequency domain characteristics (eg. total power, spectral edge frequency, wavelets) and information theory (eg. Shannon entropy, Fisher characteristics). Extracted features from each epoch are then fed into a support vector machine (SVM), a learning algorithm that has been pretrained on a training EEG dataset marked with seizure/non-seizure by an expert. The outputs of the SVM are then converted using a sigmoid function into a probability of seizure between 0 and 1. This output is then smoothed by a moving average filter and compared to a threshold. The comparison of the SVM probability output with the threshold is then converted to binary decisions for each channel, then for all channels. A custom reader displays a single probability graph (the channel of highest seizure probability) that turns from black to red when the threshold is breeched and a seizure designated. The threshold may be manually adjusted at increments of 0.1 such that the user can alter the sensitivity of the algorithm on a patient by patient basis. If for example, a patient’s record contains large amounts of artefact, the user may wish to desensitize the algorithm to prevent excess false detections, however concomitant seizure detection rates will also be affected. For detections, an annotation is recorded of the onset time, duration and channel of highest seizure probability and the annotation list can be exported as a text file for further analysis. The algorithm was updated (beta version) following a detailed analysis to incorporate modifications that reduce false detections due to prolonged EEG artefact (Temko et al., 2013). This updated version of the algorithm has recently been tested on a large dataset of EEGs from 70 babies for the purposes of performance validation on an unseen dataset (Mathieson et al., 2016b).

### 2.4 Seizure analysis

All EEGs were reviewed by a clinical physiologist (SM) and EEG seizures were annotated as the ‘gold standard’ at the start and end of seizures to give exportable text files listing seizure onset and duration times. Seizures annotated by SM were verified by a consultant neurophysiologist (RMP) by reviewing the EEG at the time of the annotation by SM. Each seizure was then analysed by SM using the criteria outlined in Table 1 and features for pre- and post-phenobarbital seizures were compared statistically. The 10 features analysed were drawn from a previous in depth analysis of seizure factors affecting automated seizure detection by ANSeR (Mathieson et al., 2016a), and were chosen for the previous study specifically with the feature extraction criteria and general functioning of the algorithm in mind but were also deemed relevant as a general methodology for quantifying seizures for the present study to assess the effects of phenobarbital on seizures. The 10 features can be grouped into 3 broad categories: ‘seizure signature’ (1–5), ‘short-term temporal context or evolution’ of seizures (6–8) and ‘seizure spatial context’ (9–10). In the ‘seizure signature’ group, seizure amplitude was measured at the midpoint of the seizure as a common reference point for measuring peak seizure amplitude which provides a measure of the ‘strength’ of the seizure and/or degree of neuronal recruitment. The rhythmicity score was scored from visual assessment of the apparent degree of rhythmicity of the seizure from second to second, to determine whether phenobarbital changes the rhythmicity of seizures over short timespans. The background EEG score provides a measure of the effect of phenobarbital on the baseline EEG at the time of the seizures. The seizure morphology at seizure onset and peak tells us whether the dominant frequency and shape of the seizure waveform at those timepoints change with phenobarbital. In the ‘short term temporal context or evolution’ group, seizure morphology change from onset to peak tells us whether seizure morphology within seizures evolves and whether phenobarbital affects this, while frequency variability (over the whole seizure) gives an indication of the amount of change or evolution of frequencies within the seizure, given that many neonatal seizures tend to slow in frequency, particularly towards the end (personal observation). A measure of seizure duration was taken to determine whether phenobarbital affects seizure length. In the ‘spatial context’ group, the number of EEG channels involved in the seizure at start and peak gives an indication of the approximate size of the seizure field at the start and peak and whether phenobarbital affects these fields. Criteria for assessing seizure discharge morphology at onset and peak were adapted from Patrizi et al. (Patrizi et al., 2003), and criteria for scoring the background EEG activity at the time of the seizure were taken from Pressler (Pressler et al., 2001).

All EEGs were then analysed by ANSeR at a sensitivity threshold of 0.3 and seizure annotations were exported as text files. In a previous validation study (Mathieson et al., 2016b), ANSeR performance was assessed across the full range of sensitivity thresholds on a cohort of 70 babies. In this study we considered a threshold range from 0.5 to 0.3 to be within a clinically acceptable range, giving seizure detection rates of 52.6–75.0% and false detection rates of 0.04–0.36 FD/h respectively. This range was proposed on the basis of a perceived expectation that a minimum of 50% seizure detection would be required by users in a clinical setting and that a false detection rate of greater than 0.5/h might be considered excessive. Given a variable threshold a given user may of course decide to set a higher sensitivity threshold if they prefer to detect more seizures at a cost of more false alarms, or vice-verse. The clinical suitability of this range is then, opinion based. The highest sensitivity threshold of 0.3 within this range was chosen for this study to highlight the highest seizure detection rate realistically achievable.

### 2.5 Statistical method

For each neonate and separately for the two time periods (pre and post-phenobarbital administration), a summary measure across seizures was calculated for each of the parameters of interest. The median was used as the summary measure for peak amplitude, seizure duration, number of EEG channels involved in seizure onset and at the peak of the seizure and frequency variability. The maximum value was the summary measure used for rhythmicity score and background EEG. Rhythmicity score and background EEG were qualitative variables with only 3 and 4 categories each, respectively. Hence, the highest category observed (maximum) for each baby was a suitable summary measure for these variables. The other variables were quantitative variables, taking on a range of possible values, and hence the median was a suitable summary measure for these variables. For the seizure detection rate, discharge morphology and discharge morphology change, the proportion was used as the summary measure. Differences between the two time periods (pre- and post-phenobarbital administration) were investigated using the Wilcoxon Signed Rank test. All statistical analyses were performed using IBM SPSS Statistics, version 20.0. All tests were two-sided and a *p*-value < 0.05 was considered to be statistically significant.

## 3 Results

### 3.1 Patients

Details of included patients are given in Table 2. All patients received a loading dose of phenobarbital (20 mg/kg) and 3 patients received a second dose of phenobarbital.

### 3.2 Comparison of pre- and post-phenobarbital seizure features and automated detection

In total 524 seizures (262 pre-phenobarbital seizures and 262 post-phenobarbital seizures) were identified and annotated on the original EEG by SM for feature analysis. Verification of these seizures by RMP showed 100% agreement.

The results of the comparison of seizure features between pre- and post-phenobarbital seizures are shown in Table 3. Post-phenobarbital seizures were of statistically lower peak amplitude than pre-phenobarbital seizures [median (interquartile range) pre-Pb: 123 μV (62.5–225) vs post-Pb: 53.5 μV (46.13–89.25)], with a drop of 56.5%, and involved fewer EEG channels at the peak of seizure [median (interquartile range) pre-Pb: 4 channels (3–8) vs post-Pb: 3 channels (1.4–4)]. Values for individual babies for median peak seizure amplitude and median number of EEG channels involved at peak of seizure pre- and post phenobarbital are given in Table 4.

**Fig. 1:**
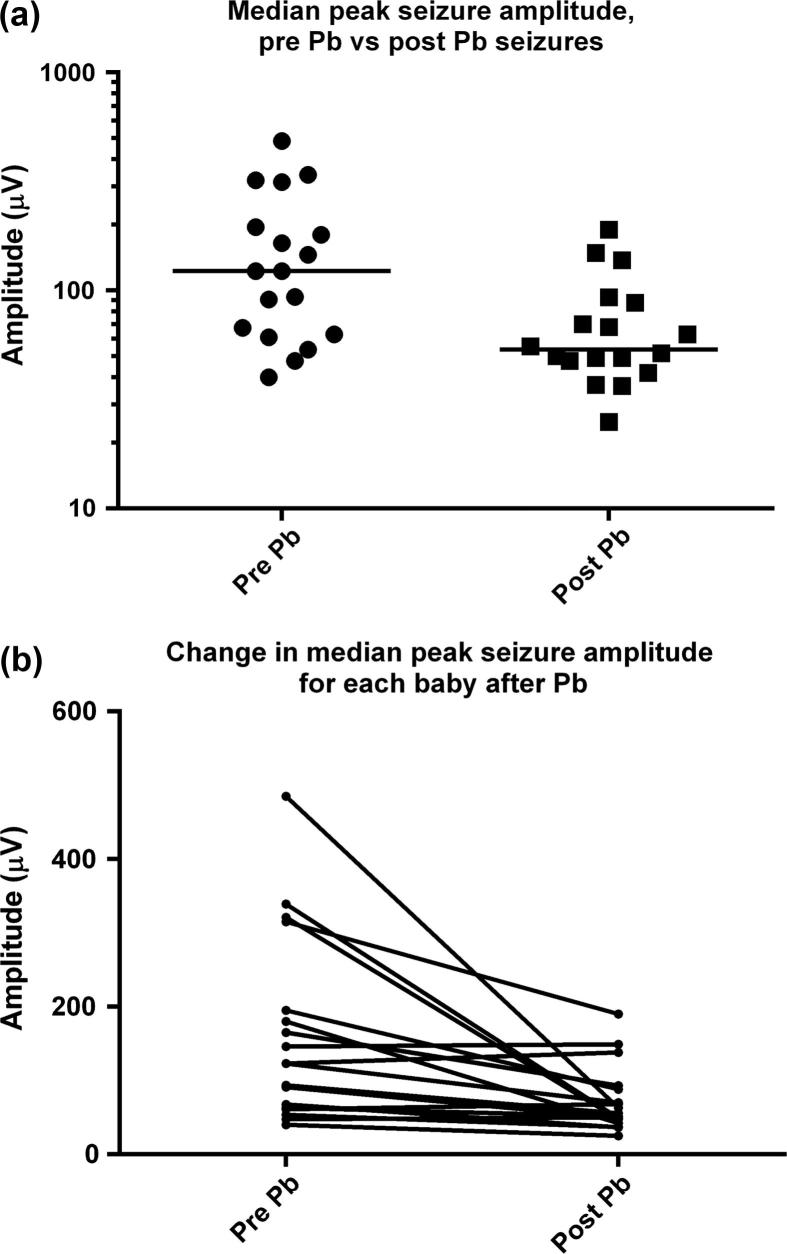
(a) Comparison of median peak seizure amplitudes for pre- and post-Pb seizures in all neonates. Black line represents group median. (b) Change in median peak seizure amplitude for each baby after Pb.

Fig. 1a shows a comparison of the pre-and post-phenobarbital median seizure amplitudes for all 18 neonates including the group median (thick black line), while Fig. 1b shows the change in amplitude for each baby after phenobarbital; seizures were reduced in amplitude after phenobarbital in 14 of 18 babies. Babies with high amplitude seizures pre-phenobarbital showed the greatest reduction in seizure amplitude.

**Fig. 2:**
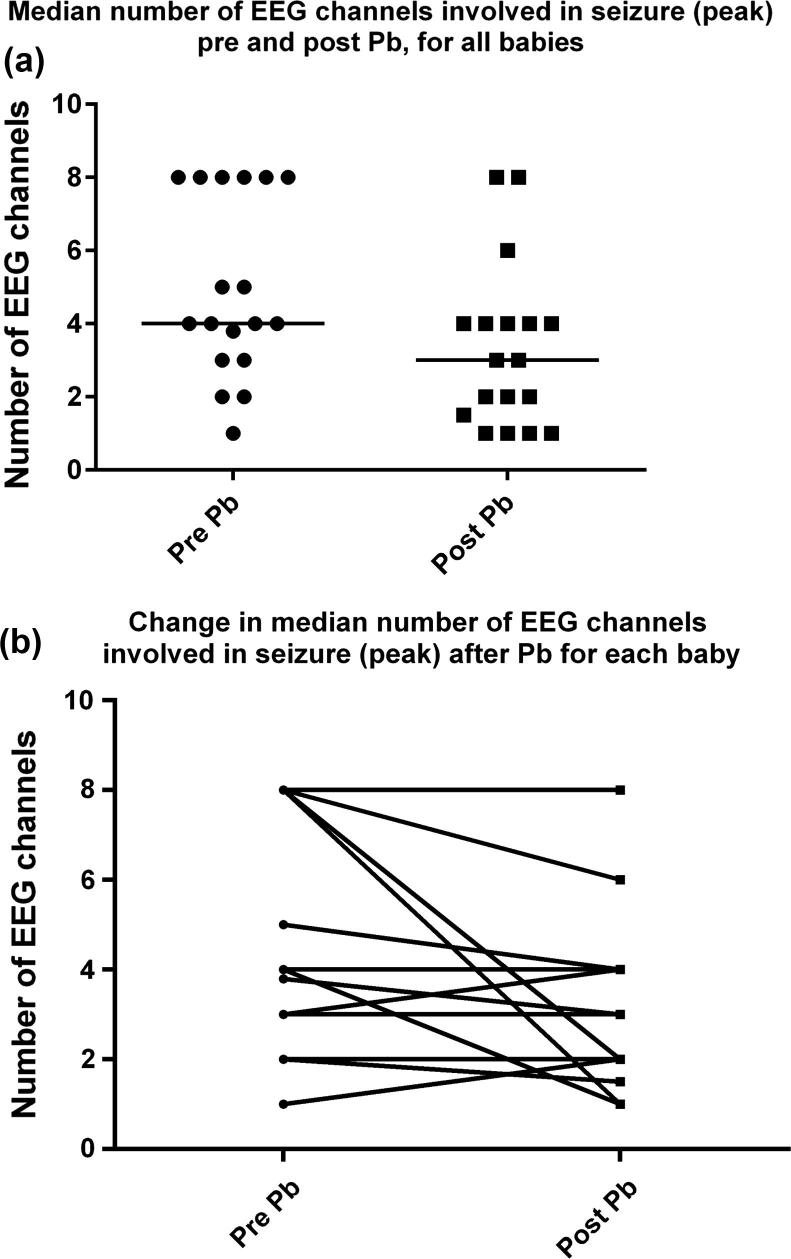
(a) Comparison of median number of EEG channels involved at peak of seizure pre- and post-Pb for all neonates. Black line represents group median. (b) Change in median number of EEG channels involved in seizure (peak) after Pb for each baby.

Fig. 2a shows the median number of EEG channels involved in pre and post-phenobarbital seizures (at the peak of seizure) for all neonates with group median (black line) and Fig. 2b shows the change in number of EEG channels involved in the seizure for each baby. A reduction in the number of channels involved was seen in 10 of 18 babies.

**Fig. 3:**
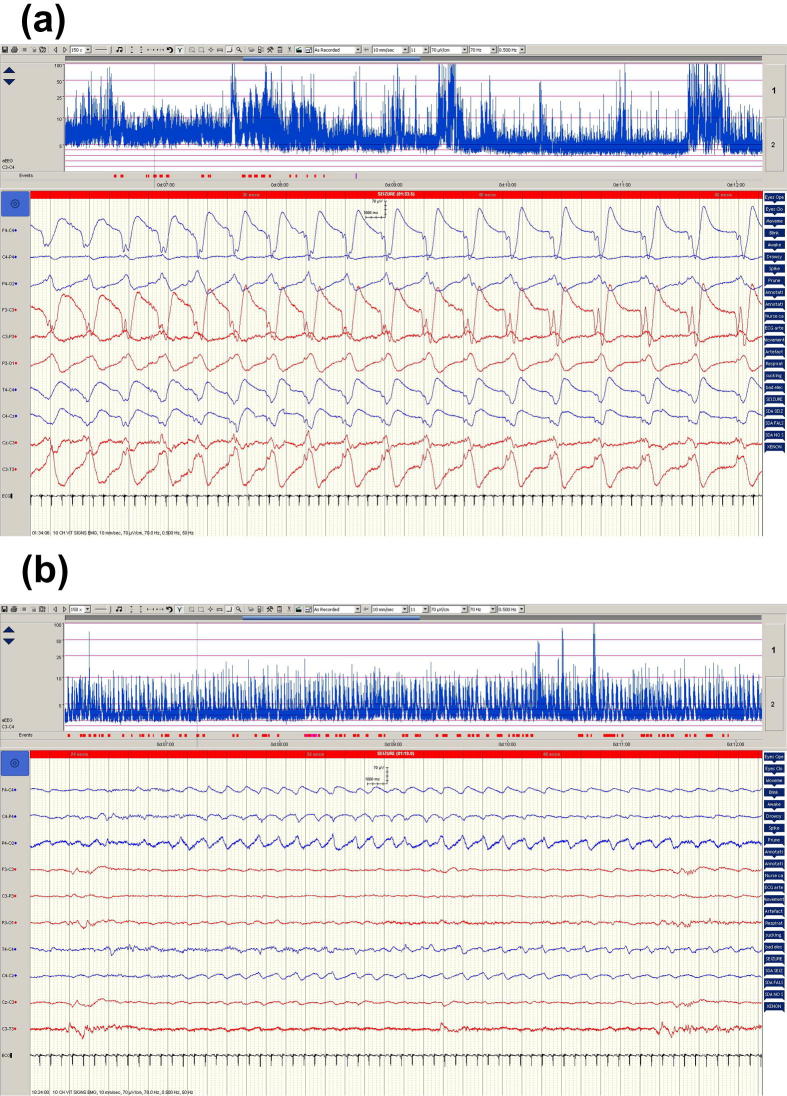
Examples of pre- and post-Pb seizures for patient 5. (a) Pre-Pb seizure and (b) post-Pb seizure.

Fig. 3 shows typical examples of pre- and post-phenobarbital seizures for patient 5 showing a drop in seizure amplitude and a reduced number of EEG channels involved post-phenobarbital treatment.

No significant differences between groups were found in seizure duration, rhythmicity, frequency variability (over the whole seizure), background EEG grade, seizure waveform morphology at the start or peak of the seizure or seizure waveform morphology change from start to peak of seizure.

**Fig. 4:**
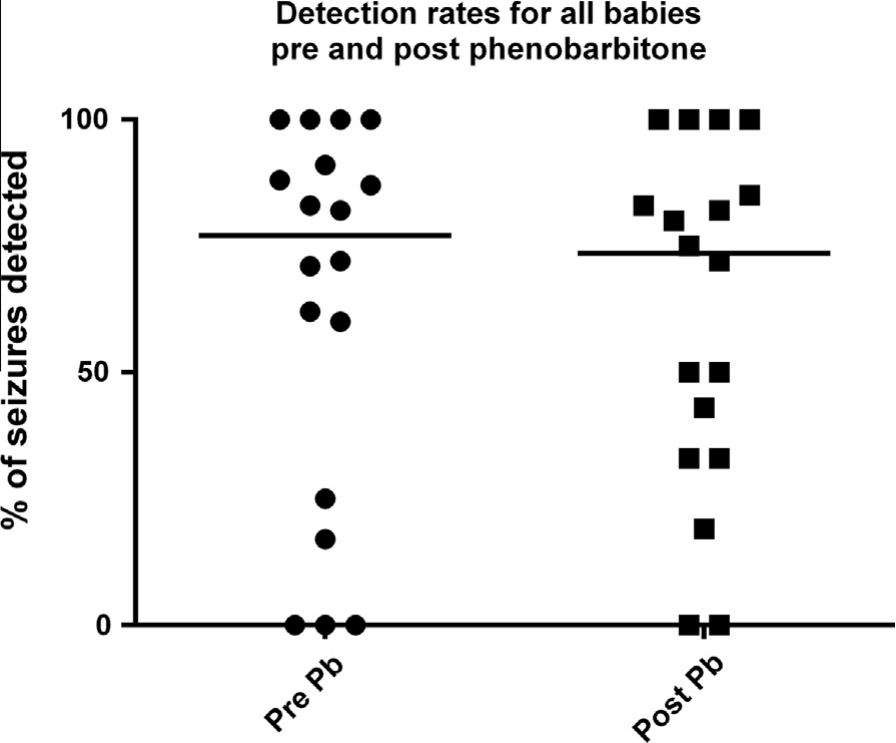
Distribution of detection rates for all neonates pre- and post-phenobarbital. Black lines indicate median values.

Fig. 4 shows seizure detection rates for all neonates pre and post-phenobarbital administration. The seizure detection rates (sensitivity threshold 0.3) were not significantly different with a median detection rate of 77% for pre-phenobarbital seizures and 73% detection rate for post-phenobarbital seizures.

### 3.3 Electroclinical uncoupling

Information regarding electroclinical uncoupling is summarised in Table 4. Information was only available for 15 of 18 babies as 3 babies (12, 13, 16) had no video available for analysis. Of these 15, 9 babies (1, 4, 5, 6, 7, 8, 11, 14, 15) had electrographic seizures both before and after phenobarbital and therefore had no electroclinical uncoupling. One baby (17) had a mixture of electroclinical and electrographic seizures both before and after phenobarbital so again, had no clear evidence of electroclinical uncoupling. This baby did not have a reduction in median seizure amplitude (pre-Pb 47.5 μV vs post-Pb 50 μV), or a reduction in the median number of EEG channels involved at the peak of seizure (pre-Pb 3.0 channels vs post-Pb 4 channels). Five babies (2, 3, 9, 10, 18) had electroclinical seizures before phenobarbital and electrographic only seizures after phenobarbital and therefore experienced electroclinical uncoupling after phenobarbital. Baby 2 had three low amplitude focal seizures before phenobarbital with subtle changes in blood pressure and heart rate, and further focal, low amplitude seizures without physiological changes after phenobarbital. There was both a drop in median seizure amplitude between pre- and post-phenobarbital seizures (pre-Pb 40 μV vs post-Pb 20 μV) and also a marked drop in the median number of channels involved at seizure peak (pre-Pb 4 channels vs post-Pb 1 channel). Babies 9, 10 and 18 all had unilateral stroke (middle cerebral artery) with clonic seizures prior to phenobarbital. None of these babies experienced large reductions in the number of EEG channels involved in seizure peak (baby 9 pre-Pb 3.8 channels vs post-Pb 3 channels, baby 10 pre-Pb 4 channels vs post-Pb 4 channels, baby 18 pre-Pb 4 channels vs post-Pb 4 channels), however all had large reductions in median peak seizure amplitude after phenobarbital (baby 9 pre-Pb 321 μV vs post-Pb 47.5 μV, baby 10 pre-Pb 195 μV vs post-Pb 88 μV, baby 18 pre-Pb 123 μV vs post-Pb 70 μV). Baby 3 had HIE with bilateral watershed infarcts with clonic seizures prior to phenobarbital. This baby had both a large reduction in median seizure amplitude after phenobarbital (pre-Pb 339 μV vs post-Pb 49 μV) and a large reduction in the number of channels involved at seizure peak (pre-Pb 8 channels vs post-Pb 1 channel).

## 4 Discussion

This study has shown that phenobarbital reduces both the amplitude and propagation of seizures. Electroclinical uncoupling of seizures is an important phenomenon as seizures may go undetected and untreated in centres without EEG monitoring. A model for electroclinical uncoupling was proposed by Glykys et al. (Glykys et al., 2009), based on observations in rat pups of a differential expression of the NKCC1 and KCC2 chloride transporters between cortical and subcortical regions, potentially rendering GABA excitatory in cortical regions and inhibitory at subcortical levels. In this scenario, cortical seizures as detected by EEG would be unaffected or even exacerbated by phenobarbital while the inhibition at the subcortical level would block their clinical expression. The findings in our study, that cortical seizures are reduced in amplitude and propagation, does not support the cortical aspect of the model proposed by Glykys, nor do studies showing that phenobarbital is effective in abolishing neonatal seizures in 30–50% of cases (Painter et al., 1999, Boylan et al., 2002, Boylan et al., 2004). It is still possible however that an increased efficacy of phenobarbital in subcortical regions relative to the cortex, as proposed by Glykys, may contribute to electroclinical uncoupling.

While one study (Boylan et al., 2002) reported an increase in seizure burden in some cases after phenobarbital administration, it is clear that seizures have a natural history, increasing, peaking and tailing off in frequency (Lynch et al., 2012) such that an increase in seizure burden after phenobarbital may simply be a consequence of this process rather than due to the anticonvulsant. This is supported by the study by Painter (Painter et al., 1999) in which phenobarbital was found to be ineffective in neonates in the phase of increasing seizure burden.

When considering the causes of electroclinical uncoupling, it is also possible that in subclinical seizures there is A) simply less involvement of the motor strip as previously suggested by Boylan et al. (Boylan et al., 2013), the area from where the most overt clinical expression of seizures originates, and/or B) if one assumes that the amplitude of a seizure in a given area roughly equates to the number of neuronal units recruited, then it may be that there is a threshold of neuronal numbers in the cortex that must be breached in order to drive subcortical circuits and in turn motor manifestations of seizures. Subclinical seizures may simply not reach this threshold due to a partial dampening of seizure activity by phenobarbital.

While this study has not addressed the specific question of the relative involvement of the cortical motor area in clinical vs subclinical seizures, the finding that overall, post-phenobarbital seizures tend to propagate less than pre-phenobarbital seizures does at least suggest that this possibility is worth investigating. A study of the relative involvement of the motor area in pre and post phenobarbital seizures would be optimised using a more comprehensive set of electrodes rather than the reduced set used here in which electrode coverage away from the motor area was quite sparse. The finding that seizures in these babies dropped 56.5% as a group in their median amplitude after phenobarbital administration also supports the second possibility.

The evidence from the babies in this study regarding electroclinical uncoupling supports these suggestions with the 1 patient who experienced some electroclinical seizures both before and after phenobarbital showing no reduction in seizure amplitude or propagation after phenobarbital, while the 5 patients with electroclinical seizures before phenobarbital and electroclinical uncoupling after phenobarbital, all experience a drop in either the propagation of the seizure or the peak amplitude of seizure, or both. It is interesting to note that the 3 stroke babies (all with middle cerebral artery infarctions) that experienced electroclinical uncoupling after phenobarbital, did not have a marked reduction in the number of channels involved at the peak of seizure, therefore there was presumably still involvement of the motor cortex in their seizures. They did however, experience marked reduction in seizure amplitude. This limited information does lend some weight to the suggestion that seizure amplitude is the dominant factor in electroclinical uncoupling.

Another important consequence of a reduction in seizure amplitude and propagation after phenobarbital is that seizures are likely to become harder to detect on visual examination of the electrographic evidence. This is true of the EEG evidence where post-phenobarbital seizures may stand out less from the background EEG, but is likely to be more of a problem when only aEEG monitoring is available. Studies by Rennie et al. (Rennie et al., 2004) and others (Shellhaas et al., 2007, Mastrangelo et al., 2013) have shown that many seizures detected on EEG are missed when reviewing the aEEG alone and that ‘missed’ seizures are often short, involve a small number of EEG channels and of low amplitude, often registering minimal deflection on the aEEG trace. These findings highlight that EEG should be used for seizure monitoring where available and that great care should be used when reviewing EEG after anti-seizure medication.

Eleven of the 18 babies in this study had HIE and underwent therapeutic hypothermia which is now a routine treatment. Cooling has been shown to reduce the seizure burden in this group (Low et al., 2012), perhaps due to the prolonged half-life of phenobarbital in cooled patients (Roka et al., 2008, Filippi et al., 2011), and/or augmenting the neuroprotective effects of hypothermia, as has been shown in a rodent model (Barks et al., 2010). Despite the positive effects of cooling, seizures remain a problem in HIE.

The main purpose of this study was to examine the effect of phenobarbital on the morphology of seizures and on the performance of our seizure detection algorithm. In a previous study (Mathieson et al., 2016a) to investigate the features of seizures affecting ANSeR detection, using the same methodology for seizure analysis but multivariate analysis, it was found that an increase in 4 features; seizure duration, amplitude, rhythmicity and number of EEG channels involved in seizure peak (propagation), were independently associated with an increased likelihood of seizure detection by ANSeR. In the present study phenobarbital did not affect seizure duration or seizure rhythmicity but did reduce seizure amplitude and propagation. However this reduction was not enough to significantly reduce ANSeR performance. It is possible that the reduction in seizure amplitude and propagation seen in our previous paper was greater due to additional treatments such as phenytoin and midazolam.

These results suggest that the performance of ANSeR is robust to the effects of phenobarbital on neonatal seizures and users should not need to adjust the sensitivity threshold after phenobarbital administration. One limitation of this study is that only the effects of phenobarbital only were tested while the effect of other second-line anti-seizure medications including phenytoin, midazolam and others, were not. As suggested above, it may be that these second line medications do affect automated seizure detection and is an area for further study, however teasing out the effects of particular drugs on seizures with patients on multiple anti-seizure medications is problematic.

## 5 Conclusion

The main purpose of this study was to examine the effect of phenobarbital on the morphology of neonatal seizures, and on the performance of our automated seizure detection system. We have shown that phenobarbital reduces the amplitude and propagation of seizures but ANSeR performance is unaffected by these changes.

## Figures and Tables

**Table 1:** Seizure features analysed.

Variable group	Variable	Measurement type: quantitative/visual analysis	Measurement unit	Method/category	Purpose/comment
Seizure signature	Seizure amplitude at peak of seizure	Quantitative	μV^2^	Measure peak to trough using graticule on highest amplitude discharge at midpoint of seizure	To quantify the maximum seizure amplitude
Seizure signature	Rhythmicity score	Visual	Number	1 = Significant dysrhythmia	Visual score of how rhythmicity/frequency appears to change from second to second over the seizure
				2 = Minimal dysrhythmia	
				3 = Highly rhythmic	
Seizure signature	Background EEG score at time of seizure	Visual	Number	1 = Normal/mildly abnormal, continuous EEG	To highlight context in which seizure are detected/not detected
				2 = Moderate abnormality. IBI < 10 s	
				3 = Severe abnormality, IBI 10–60 s	
				4 = Inactive, background < 10 μV, IBI > 60S	
				⁎See below	
Seizure signature	Seizure morphology at onset	Visual	Category	1 = Rhythmic discharges of delta (RDD)	To categorize dominant morphology of seizure discharge at onset
				2 = Rhythmic discharges of theta (RDT)	
				3 = Rhythmic discharges of alpha (RDA)	
				4 = Spikes (S) or sharp waves (SH)	
				5 = Sharp wave and slow wave (SH + W) complexes or spike and wave complexes (SP + W)	
				⁎⁎See below	
Seizure signature	Seizure morphology at peak of seizure	Visual	Category	As above	To categorize dominant morphology of seizure discharge at peak (middle) of seizure
Short-term temporal context or evolution	Seizure duration	Quantitative	Seconds	Duration derived from SM annotations of start/end of seizure.	To quantify seizure duration
Short-term temporal context or evolution	Frequency variability (over whole seizure)	Quantitative	SD (Hertz)	Using frequency graticule calculate discharge frequency at:	To derive an estimate of the degree of frequency variability over the span of the seizure.
				A = Start frequency (first 5 s)	
				B = Peak frequency (mid seizure)	
				C = Final frequency (last 5 s)	
				Frequency variability = Standard deviation A:C	
Short-term temporal context or evolution	Seizure morphology change from onset to peak	Quantitative	Binary Y/N	Comparison of seizure morphology at start and peak	To assess change/variability of seizure morphology within seizure
Spatial context	Number of EEG channels involved at onset of seizure	Visual	Number	Count of number of EEG channels showing seizure discharges	To estimate the size of the seizure field at the start of the seizure
Spatial context	Number of EEG channels involved at peak of seizure	Visual	Number	Count of number of EEG channels showing seizure discharges	To estimate the size of the seizure field at the peak of the seizure

⁎ From Pressler et al. (2001).

⁎⁎ Adapted from Patrizi et al. (2003).

**Table 2:** Aetiology, number of seizures analysed and anticonvulsants for patients included in the study. Pb: phenobarbital; na: not administered; HIE: hypoxic ischaemic encephalopathy.

Patient number	Aetiology	Seizure number studied pre/post Pb	1st dose Pb (mg/kg)	2nd dose Pb (mg/kg)
1	HIE grade 2	7/7	20	na
2	HIE grade 2	3/3	20	na
3	HIE grade 2	6/6	20	na
4	HIE grade 3	8/8	20	na
5	HIE grade 3	25/25	20	na
6	Meningitis	3/3	20	na
7	HIE grade 3	66/66	20	10
8	Contusion post forceps	1/1	20	na
9	Stroke	2/2	20	na
10	Stroke	11/11	20	na
11	HIE grade 3	4/4	20	na
12	Focal lesion	1/1	20	na
13	HIE grade 2	5/5	20	na
14	HIE grade 3	1/1	20	na
15	HIE grade 3	68/68	20	10
16	Stroke	6/6	20	na
17	HIE grade 3	6/6	20	na
18	Stroke	39/39	20	10
	Total	262/262		

**Table 3:** Results of comparison of seizure features pre- and post-phenobarbital.

Features	Summary measure	Pre-Pb seizures. [Median (IQR)]	Post-Pb seizures. [Median (IQR)]	*p* value⁎
Peak amplitude (μV)	Median	123 (62.5–225)	53.5 (46.13–89.25)	0.001
Seizure duration (s)	Median	103 (69.13–185.25)	135 (62.63–235)	0.420
Number of EEG channels involved in seizure onset (*n*)	Median	4 (2.38–4.25)	3 (1–4)	0.068
Number of EEG channels involved at peak of seizure (*n*)	Median	4 (3–8)	3 (1.38–4)	0.018
Frequency variability (over whole seizure) Hz	Median	0.25 (0.07–1.64)	0.24 (0.06–1.85)	0.868
Rhythmicity score (1–3)	Maximum	3 (2–3)	3 (2–3)	0.317
Background EEG (1–4)	Maximum	1.5 (1–4)	1.5 (1–3.25)	0.317
Seizure detection rate	Proportion	0.77 (0.23–0.93)	0.73 (0.33–0.89)	0.730
Changed morphology	Proportion	0.45 (0.11–0.88)	31 (0.0–0.67)	0.310

⁎ From Wilcoxon Signed Rank test. N.B. Comparisons of discharge morphology (RDD, RDT, RDA, SP/SH, SP + W/SH + W) at start and peak of seizure are omitted, *p* > 0.05 for all comparison.

**Table 4:** Evidence of electroclinical uncoupling, seizure amplitudes and number of EEG channels involved at peak of seizure before and after phenobarbital for individual babies. NVD no video data, EG electrographic, EC electroclinical.

Study ID	Aetiology	Seizure type pre-Pb	Seizure type post-Pb	Electroclinical uncoupling?	Median peak seizure amplitude pre-Pb (μV)	Median peak seizure amplitude post-Pb (μV)	Median number of EEG channels involved in seizure peak pre-Pb	Median number of EEG channels involved in seizure peak post-Pb
12	Focal lesion	NVD	NVD	Unknown	91	49	4	1
13	HIE grade 2	NVD	NVD	Unknown	123	138	2	2
16	Stroke	NVD	NVD	Unknown	93.5	55.5	5	4
1	HIE grade 2	EG	EG	NO	485	63	8	1
4	HIE grade 3	EG	EG	NO	146	149	8	8
5	HIE grade 3	EG	EG	NO	180	42	8	6
6	Meningitis	EG	EG	NO	165	93	8	8
7	HIE grade 3	EG	EG	NO	67.5	36.5	5	4
8	Contusion post forceps	EG	EG	NO	61	68	1	2
11	HIE grade 3	EG	EG	NO	63	51.5	2	1.5
14	HIE grade 3	EG	EG	NO	315	190	8	2
15	HIE grade 3	EG	EG	NO	53.5	37	3	3
17	HIE grade 3	EC/EG	EC/EG	NO	47.5	50	3	4
2	HIE grade 2	EC	EG	YES	40	20	4	1
3	HIE grade 2	EC	EG	YES	339	49	8	1
9	Stroke	EC	EG	YES	321	47.5	3.8	3
10	Stroke	EC	EG	YES	195	88	4	4
18	Stroke	EC	EG	YES	123	70	4	4
